# Reliability and validity of the functioning assessment short test for older adults with bipolar disorder (FAST-O)

**DOI:** 10.1186/s40345-020-00193-2

**Published:** 2020-10-02

**Authors:** Melis Orhan, Nicole Korten, Ralph Kupka, Patricia van Oppen, Max Stek, Eduard Vieta, Sigfried Schouws, Wouter van Ballegooijen, Annemiek Dols

**Affiliations:** 1 Department of Old Age Psychiatry, GGZ inGeest, Amstelveenseweg 589, 1081JC Amsterdam, The Netherlands; 2 Department of Psychiatry, Amsterdam Public Health Research Institute, VU University Medical Center, Amsterdam, The Netherlands; 3 Hospital Clinic, University of Barcelona, IDIBAPS, CIBERSAM, Barcelona, Catalonia Spain; 4 Neuroscience Campus, VUMC, Amsterdam, The Netherlands

**Keywords:** Older, Bipolar disorder, Daily functioning, Instrument, Reliability, Validity

## Abstract

**Background:**

Many frequently used instruments fail to assess psychosocial functioning in patients with bipolar disorder. The Functioning Assessment Short Test (FAST) was developed in order to tackle this problem and to assess the main functioning problems experienced by patients with bipolar disorder. However, the original FAST is not fully applicable in older adults due to the domain of occupational functioning. The aim of our study was to validate an adapted version for Older adults (FAST-O) in a group of older adults with bipolar disorder (OABD).

**Methods:**

88 patients aged 50 years and over diagnosed with bipolar disorder were included. We adapted the items in the area of “work-related functioning” of the FAST into items assessing “societal functioning”. Several measurements were conducted in order to analyse the psychometric qualities of the FAST-O (confirmatory factor analysis for internal structure, Cronbach’s alpha for internal consistency, Spearman’s rho for concurrent validity, Mann–Whitney U test for discriminant validity).

**Results:**

Mean age in the study sample was 65.3 (SD = 7.5) and 57.3% was female. The internal structure was most similar to the internal structure of the original FAST. The internal consistency was excellent (Cronbach’s alpha = .93). The concurrent validity when correlated with the Social and Occupational Functioning Assessment Scale was low, but significant. The FAST-O was also able to distinguish between euthymic and symptomatic OABD patients.

**Conclusions:**

The FAST-O has strong psychometric qualities. Based on our results, we can conclude that the FAST-O is a short, efficient solution in order to replace global rating scales or extensive test batteries in order to assess daily functioning of older psychiatric patients in a valid and reliable manner.

## Background

Bipolar disorder (BD) is a severe, episodic, lifelong mood disorder that is defined by episodes of mania or hypomania alternating or occurring concomitantly with depressive episodes, and euthymic phases (Fagiolini et al. 2013). Although the prevalence of BD seems to decline with age, still 8–10% of psychiatric inpatients over age 55–60 are diagnosed with BD (Depp and Jeste 2004). Contrary to the traditional view that individuals with BD are asymptomatic between episodes and return to normal functioning, recent studies have revealed a much less optimistic picture (Pope et al. 2007). Many patients with BD in clinical remission experience residual mood symptoms, social dysfunction, cognitive impairment and stigma (Samalin et al. 2016). As a result, 30–60% of adult patients with BD suffer from poor psychosocial functioning (MacQueen et al. 2008). Functioning is a complex construct that involves many interactions and activities in personal, occupational, and recreational contexts (Zarate et al. 2002; Tohen et al. 2000). Therefore, formulating a clear definition is often a challenge. The ICF identifies three levels of human functioning: functioning at the level of body or body part, the whole person, and the whole person in a social context. Disability therefore involves dysfunctioning at one or more of these same levels: impairments, activity limitations and participation restrictions (World Health Organization 2002). In older age BD (OABD), functioning may become further limited as a result of various factors, such as a decreasing social network size and reduced mobility (Liempt et al. 2016). Social functioning is also related to cognitive functioning (Orhan et al. 2018), which is worse in OABD patients compared with healthy controls (Schouws et al. 2012).

Due to a lack of easy-to-use instruments, patients need to undergo an extensive battery of tests to fully assess all the relevant factors regarding daily functioning, which is time-consuming for both patient and therapist. Besides these extensive batteries, the currently most frequently used measures to assess daily functioning are global rating scales. However, these fail to distinguishing clinical and functional recovery (Berns et al. 2007) and do not include all areas of daily functioning. To tackle this problem, experts of the Bipolar Disorder Program (Barcelona, Spain) identified six main areas of problems experienced by patients with BD, including autonomy, occupational functioning, cognitive functioning, financial issues, interpersonal relationships, and leisure time (Depp and Jeste 2004). Subsequently, these findings were used to design the Functioning Assessment Short Test (FAST); a quick and easily administrable instrument for the clinical evaluation of daily impairments presented by patients suffering from BD (Rosa et al. 2007). However, in older adults the FAST may not be fully applicable given the questions in the domain of occupational functioning.

In the original FAST manual (Rosa et al. 2007), some suggestions were proposed to alter the items on the occupational scale for patients whose occupational functioning cannot be rated. In line with these suggestions, we adapted the FAST for the OABD population into the FAST-O, the Functioning Assessment Short Test for Older adults (FAST-O). Hereby the domain of occupational functioning was altered slightly and items on the original FAST were translated to Dutch. The FAST has been validated in several languages for adult patients with BD and these studies all show similar positive results regarding its reliability and validity (Barbato et al. 2013; Suominen et al. 2015; Zhang et al. 2018). However, to date the FAST has not been validated in an OABD sample. The aim of this study was therefore to validate the Dutch version of the FAST in the assessment of functional impairments in a group of OABD.

## Methods

### Study sample

The sample of this cross-sectional study included OABD patients from the Amsterdam mental health catchment area. Data were used from the Dutch Older Bipolars (DOBi) dynamic cohort study (Dols et al. 2014). A computerized search into the electronic record-keeping system of the Mental Health Organization identified all patients aged ≥ 50 in contact with health services, who had any registered diagnosis that could indicate BD. Medical records were screened by a psychiatrist for exclusion criteria: not being able to give written informed consent, not being able to communicate in Dutch, mental retardation (IQ < 70), poor cognitive performance (MMSE < 18), or a highly unstable psychiatric condition. Inclusion was possible when participants were clinically diagnosed by their treating therapist with bipolar I disorder, bipolar II disorder, or bipolar disorder not otherwise specified. We included 88 patients in total. The study was approved by the Medical Ethics Committee of the VU University Medical Center, Amsterdam, Netherlands.

### Demographic and clinical characteristics

Sociodemographic data were obtained by interview. Diagnosis, type of bipolar disorder, and age at first depressive or (hypo)manic episode were established by the Mini-International Neuropsychiatric Interview Plus (MINI-plus (Sheehan et al. 1997)). The number of psychiatric admissions was obtained by clinical interview. Current mania symptoms were assessed through the Young Mania Rating Scale (YMRS (Young et al. 1978)). The YMRS is scored on a scale from 0 to 60 with scores ≥ 12 indicating clinically relevant (hypo) mania. Current symptoms of depression were measured by the Centre for Epidemiologic Studies Depression Scale (CES-D (Radloff 1977)), a 20-item self-report scale measuring severity of depressive symptoms during the previous week, with scores ranging from 0 to 60. Scores ≥ 16 indicate clinically relevant depression. To assess psychosocial functioning, the Social and Occupational Functioning Assessment Scale (SOFAS (American Psychiatric Association 2000)) was conducted. The SOFAS scores the level of global social functioning in the previous week, as estimated by patients’ treating psychiatrist. The achieved score is a global rating of current social functioning ranging from 1 to 100, with lower scores indicating lower social functioning and scores ≥ 90 suggesting no social impairments. All tests were administered by trained research assistants.

### Functioning Assessment Short Test (FAST)

The FAST was originally developed to assess the main problems in functioning experienced by psychiatric patients, particularly BD patients (Rosa et al. 2007). The FAST was translated in Italian (Moro et al. 2012), Portuguese (Cacilhas et al. 2009), Turkish (Aydemir and Burak 2012), Finnish (Suominen et al. 2015) and Chinese (Zhang et al. 2018). It comprises 24 items divided into six aspects of daily functioning: autonomy, occupational functioning, cognitive functioning, financial issues, interpersonal relationships, and leisure time. The FAST is an interviewer-administered instrument, and is designed to be conducted by a trained clinician. The studied time frame refers to the last 15 days before the assessment. In order to make the instrument more applicable for the older age population, we have replaced the domain of occupational functioning by the domain of societal functioning and therefore altered three items. We altered item 5 from “holding down a paid job” to “holding down meaningful daytime activities”, item 7 from “working in the field in which you were educated” to “working (paid of voluntary), including taking care of grandchildren or taking care of a family member” and item 8 from “occupational earnings” to “earnings (from work or payment/retirement fund)”. All items are rated using a 4-point scale, 0 = no difficulty, 1 = mild difficulty, 2 = moderate difficulty and 3 = severe difficulty. The overall score is the sum of all items, whereby higher scores indicate more serious impairment. The number of items in the FAST-O was equal to the number of items in the original FAST. See Table 1 for the FAST-O. The FAST-O is easy to administer and can be administered by any trained health care professional. The time it takes to administer the FAST-O is around 15 min.

**Table 1:** The Functioning Assessment Short Test for Older adults (FAST-O)

		To what extent is the patient experiencing difficulties in the following aspects?			
		No difficulty	Mild difficulty	Moderate difficulty	Severe difficulty
	Autonomy				
1	Taking responsibility for a household	^0^	^1^	^2^	^3^
2	Living on your own	^0^	^1^	^2^	^3^
3	Doing the shopping	^0^	^1^	^2^	^3^
4	Taking care of yourself (physical aspects, hygiene)	^0^	^1^	^2^	^3^
	Societal functioning				
5	Maintaining meaningful daily activities	^0^	^1^	^2^	^3^
6	Accomplishing tasks as quickly as necessary	^0^	^1^	^2^	^3^
7	Working (in a paid or voluntary job), including taking care of grandchildren and informal care	^0^	^1^	^2^	^3^
8	Income (occupational earnings or income from retirement)	^0^	^1^	^2^	^3^
9	Managing the expected work load or other tasks	^0^	^1^	^2^	^3^
	Cognitive functioning				
10	Ability to concentrate on a book, film	^0^	^1^	^2^	^3^
11	Ability to make mental calculations	^0^	^1^	^2^	^3^
12	Ability to solve a problem adequately	^0^	^1^	^2^	^3^
13	Ability to remember newly-learned names	^0^	^1^	^2^	^3^
14	Ability to learn new information	^0^	^1^	^2^	^3^
	Financial issues	^0^	^1^	^2^	^3^
15	Managing your own money	^0^	^1^	^2^	^3^
16	Spending money in a balanced way	^0^	^1^	^2^	^3^
	Interpersonal relationships				
17	Maintaining a friendship or friendships	^0^	^1^	^2^	^3^
18	Participating in social activities	^0^	^1^	^2^	^3^
19	Having good relationships with people close to you	^0^	^1^	^2^	^3^
20	Living together with your family	^0^	^1^	^2^	^3^
21	Having satisfactory sexual relationships	^0^	^1^	^2^	^3^
22	Being able to defend your interests	^0^	^1^	^2^	^3^
	Leisure time				
23	Doing exercise or participating in sport	^0^	^1^	^2^	^3^
24	Having hobbies or personal interests	^0^	^1^	^2^	^3^

### Statistical analyses

Data were analysed using the Statistical Package of the Social Sciences (version 24.0, SPSS Inc., Chicago, IL, USA) and R (version 3.5.3). Internal consistency was analyzed using Cronbach’s alpha for the total scale and for each subscale. Concurrent validity was assessed by calculating Spearman’s rho between total FAST scores and SOFAS scores. The SOFAS was chosen as an instrument to investigate concurrent validity, since it is often used as a measurement for global social functioning in mental disorders. Validity as a discriminant measure to detect differences in FAST scores between euthymic and symptomatic patients was also assessed. This was done by dividing patients by their symptom severity scores (YMRS & CES-D scores) into a euthymic and a symptomatic group. Symptomatic patients were identified by having scores above cut-off on the YMRS and CES-D, respectively equal and above 12 and 16. A Mann–Whitney U test was used to evaluate whether the FAST total scores were sensitive to the severity of symptoms. The internal structure of the FAST was studied by conducting a confirmatory factor analysis with a number of fixed factors based on the internal structure of the original FAST (Berns et al. 2007). Several confirmatory factor analysis (CFA) models were estimated, based on previously estimated models in other studies studying the internal structure of the FAST. All estimations were conducted in *R* (Aydemir and Burak 2012). We used the comparative fit index (CFI), Tucker-Lewis index (TLI), Akaike (AIC), Bayesian (BIC) and Root Mean Square Error of Approximation (RMSEA) as fit indices.

## Results

### Study sample

Summary of the demographic and clinical characteristics is shown in Table 2. Participants had a mean age of 65.3 (*SD* = 7.5), 57.3% was female and 42.7% was male. Of all participants, 76% had the Dutch nationality. Level of education was divided into three levels: low, medium and high. For 49.4%, the level of education was high. Of all participants, 30.5% was still in active paid employment. The 69.5% that was not in active paid employment was divided into 32.9% that was retired and 36.6% that was unemployed at the time of testing. The 69.5% that was not in active paid employment at the time of testing, was divided into 32.9% that was retired and 36.6% that was unemployed at the time of testing. Participants had an average of 14.8 (*SD *= 42.6) self-reported manic and 19.9 depressive episodes (*SD *= 39.7). The mean SOFAS score was 64.3 (*SD* = 14.8), indicating that our group had mild impairments in social functioning. Mean YMRS score was 3.1 (*SD* = 3.8) and mean CES-D score was 13.4 (*SD* = 11.8), indicating that on average, no significant mood symptoms were present at the time of investigation. 36.1 percent of all participants had a score of 16 or higher on the CES-D, indicating a clinically relevant depression. 4.5 percent of all participants had a score of 12 or higher on the YMRS, indicating a clinically significant (hypo)mania. Participants had an average score of 15.9 (*SD *= 13.8) on the FAST-O, with scores ranging from 0 to 68. Average scores on each subscale are found in Table 2.

**Table 2:** Demographic and clinical variables of the study sample

Variable	
Age, M(SD), range	65.33 (7.45), 51.3–86.8
Gender, female, % (n)	57.3 (47)
Nationality, Dutch, % (n)	86.4 (76)
Level of education, high % (n)	50.4 (41)
In active paid employment, yes, % (n)	30.5 (25)
Number of depressive episodes, M (SD), range	19.89 (42.57), 1–300
Number of manic episodes, M (SD), range	14.83 (39.7), 1–300
CES-D, M (SD), range	13.35 (11.84), 0–51
YMRS, M (SD), range	3.09 (3.78), 0–18
SOFAS, M (SD), range	64.31 (14,84), 40–100
FAST-O	
Total score, M (SD), range	15.93 (13.84), 0–68
Autonomy score, M (SD), range	1.88 (2.80), 0–13
Societal functioning score, M (SD), range	3.3 (3.9), 0–19
Cognitive functioning score, M (SD), range	5.29 (4.18), 0–20
Interpersonal relationships score, M (SD), range	4.58 (4.32), 0–18
Leisure time score, M (SD), range	1.39 (1.73), 0–7

*M* mean, *SD* standard deviation, *CES-D* Centre for Epidemiologic Studies Depression Scale, *YMRS* Young Mania Rating Scale, *SOFAS* Social and Occupational Functioning Assessment Scale, *FAST-O* Functioning Assessment Short Test for Older adults

### Psychometrics

#### Internal structure

In order to compare the internal structure of the FAST-O, we conducted multiple confirmatory factor analyses according to the factor analyses conducted in earlier studies on different versions of the FAST (Berns et al. 2007; Moro et al. 2012; R Core Team). The proposed model that showed the best fit when compared to our FAST-O, was the model that was also found in the original FAST article (Berns et al. 2007). Results are shown in Table 3. This study determined a five-factor structure, whereby it was observed that social functioning and interpersonal relationships were loading on the same factor. Concerning our internal structure, the CFI and TLI were respectively 0.88 and 0.86, indicating a medium fit. The Root Mean Square Error of Approximation (RMSEA) was 0.083 with a 90% CI 0.075 until 0.105.

**Table 3:** Factor loadings of the items of the FAST-O

FAST	Autonomy factor	Psychosocial factor	Cognitive factor	Financial factor	Interpersonal/Leisure factor
Item 1	0.766				
Item 2	0.749				
Item 3	0.877				
Item 4	0.841				
Item 5		0.775			
Item 6		0.845			
Item 7		0.750			
Item 8		0.652			
Item 9		0.809			
Item 10			0.718		
Item 11			0.591		
Item 12			0.836		
Item 13			0.699		
Item 14			0.823		
Item 15				0.934	
Item 16				0.901	
Item 17					0.778
Item 18					0.767
Item 19					0.819
Item 20					0.675
Item 21					0.453
Item 22					0.664
Item 23					0.605
Item 24					0.700

#### Reliability

Internal consistency was measured by Cronbach’s alpha. For the total scale, Cronbach’s alpha was .93 indicating an excellent internal consistency. No individual items can be deleted to attain a higher Cronbach’s alpha. When looking at the different domains, a high internal consistency was found for autonomy (Cronbach’s alpha = .84), with all inter-item correlations > .50. A high internal consistency was also found for the domain of social functioning (Cronbach’s alpha = 0.85), with all inter-item correlations > .36. This was also found for cognitive functioning (Cronbach’s alpha = .83), with a lowest inter-item correlation of .39. For financial functioning was an excellent internal consistency found (Cronbach’s alpha = .92), with an inter-item correlation of .85. The domain of interpersonal functioning showed a high internal consistency of .82, with a lowest inter-item correlation of .18. The last domain of leisure time had a medium internal consistency with a Cronbach’s alpha of .62, and an inter-item correlation of .46.

#### Concurrent validity

Concurrent validity based on functional impairment according to the SOFAS scale showed a low significant correlation between SOFAS scores and FAST-O scores (Spearman’s rho = −.33; *p* < .01).

#### Discriminant validity

A Mann–Whitney U test was used as a discriminant measure to explore the difference in scores between euthymic patients and symptomatic patients. The Mann–Whitney U test was significant (*p* < .01), indicating that scores on the FAST-O are different between symptomatic and euthymic patients. By calculating the ROC curve, we further explored the discriminant capacity between symptomatic and euthymic patients. The area under the curve was 0.77 (95% CI .65–90; p < .01), indicating a good capacity. Also was observed that a score above 9.5 the best balance obtains between sensitivity (87%) and specificity (74%). In the original FAST (Rosa et al. 2007) similar scores were found, where the best balance was obtained between sensitivity (72%) and specificity (87%) with a score above 11.

## Discussion

The aim of this study was to study the psychometric qualities of the FAST-O in an OABD population. We found that the FAST-O proved to be a reliable and valid measure, when adapting the items to an OABD patient sample. The instrument showed an excellent internal consistency and was successful in discriminating between symptomatic and euthymic patients. The concurrent validity was significant. The internal structure of the FAST-O showed a satisfactory fit when compared to the original FAST. Our results indicate that the FAST-O can be used as a quick and easy way to assess daily functioning in OABD patients.

Given the lack of short, practical instruments to assess daily functioning in OABD, we altered the original FAST with regard to the area of work for the use in an older patient population. The original FAST (Rosa et al. 2007) was already well-studied in several languages (Suominen et al. 2015; Zhang et al. 2018; Moro et al. 2012; Cacilhas et al. 2009; Aydemir and Burak 2012) and studies investigating psychometric qualities when used in the adult population showed positive results. In previous studies, the correlation between the FAST and the SOFAS scale was studied to assess the concurrent validity indicating a good concurrent validity. Our correlation coefficient was low in comparison with correlation coefficients found in other studies. This difference may be due to the fact that the SOFAS scale is a rating scale based on functioning in social, but also in occupational domains thereby more suited for working age adults. In our sample, only 30.5% was still in active paid employment and the SOFAS is therefore not completely suitable for these participants. The retirement age in the Netherlands is currently 67 but relatively good social networks (still) exist for those leaving a paid job before reaching this official retirement age. Also, the SOFAS assesses impairments in social and occupational functioning that are a result of the mental disorder. In the FAST-O, the term occupational functioning is replaced by societal functioning thereby shifting the focus of the rated area. Thereby, the FAST-O does not make an distinction between problems in daily functioning as a direct result of the mental health problems, but sketches a more global view of daily functioning. The FAST-O and the SOFAS therefore measure different concepts. However, we decided to draw a comparison with the SOFAS, since it is currently the most frequently used rating scale in mental health care to indicate daily functioning.

When looking into the internal structure of the FAST-O, we found that the adjusted items have high factor loadings on the same five factor as the corresponding items in the original FAST (Rosa et al. 2007). This indicates that the items that we altered for use in the older patient population resemble the same internal structure as the original items. All in all, the FAST-O has proven to be a well-suited alternative to more extensive assessment batteries in order to assess daily functioning.

In our study, we included patients of 50 years and over and the majority of our group was not in active paid employment. However, the items are also applicable to populations where a part of the population is still in active paid employment. But since the items are more applicable for patients who are not in active paid employment, we recommend using this instrument for these patients. In populations where the majority is still in active employment, we recommend using the original FAST (Rosa et al. 2007).

Our study has several limitations. First, we did not take into account different age and ethnic groups nor responsiveness over time in order to draw a conclusion about the ecological validity of the FAST-O. We also did not take into account a measure of test–retest reliability. Finally, we used a relatively small sample with light to moderate symptoms. Therefore, our results should be interpreted with caution. Still, our study also has several strong points. Until now, no other short instrument has been developed for the assessment of daily functioning in the older patient population, and our results are promising. The authors of the original FAST article published another study in which they determined specific cut-off points for the FAST (Bonnín et al. 2018). In line with the work of these authors, future research should therefore focus on investigating specific cut-off points for the FAST-O in order to draw more standardized conclusions about the current level of daily functioning.

## Conclusions

In conclusion, the FAST-O has good psychometric qualities and therefore shows good validity and reliability. The FAST has already been extensively studied in the adult population, with good results in several languages. Adapted as FAST-O, it can be used in older patient populations to assess daily functioning in a practical and time-efficient manner.

## Data Availability

All data generated or analysed during the current study are available from the corresponding author on reasonable request.

## Abbreviations

FAST: Functional Assessment Short Test
FAST-O: Functional Assessment Short Test for Older adults
BD: Bipolar disorder
OABD: Older age bipolar disorder
MINI-plus: Mini-International Neuropsychiatric Interview Plus
YMRS: Young Mania Rating Scale
CES-D: Centre for Epidemiologic Studies Depression Scale
SOFAS: Social and Occupational Functioning Assessment Scale
SPSS: Statistical Package of the Social Sciences
CFA: Confirmatory factor analysis
CFI: Comparative fit index
TLI: Tucker–Lewis index
AIC: Akaike
BIC: Bayesian
RMSEA: Root Mean Square Error of Approximation
